# Exosomal miRNAs as a Promising Source of Biomarkers in Colorectal Cancer Progression

**DOI:** 10.3390/ijms23094855

**Published:** 2022-04-27

**Authors:** Tahani Bakhsh, Safiah Alhazmi, Najla Ali Alburae, Ali Farsi, Faisal Alzahrani, Hani Choudhry, Ahmed Bahieldin

**Affiliations:** 1 Department of Biology, Faculty of Science, Jeddah University, Jeddah 21589, Saudi Arabia; 2 Department of Biological Sciences, Faculty of Science, King Abdulaziz University, Jeddah 21589, Saudi Arabia; shalhazmi@kau.edu.sa (S.A.); nalbourai@kau.edu.sa (N.A.A.); abmahmed@kau.edu.sa (A.B.); 3 Department of Surgry, Faculty of Medicine, King Abdulaziz University, Jeddah 21589, Saudi Arabia; alihmfarsi@gmail.com; 4 King Fahd Medical Research Center, Embryonic Stem Cells Unit, Department of Biochemistry, Faculty of Science, King AbdulAziz University, Jeddah 21589, Saudi Arabia; faahalzahrani@kau.edu.sa; 5 Centre of Artificial Intelligence in Precision Medicines, King Abdulaziz University, Jeddah 21589, Saudi Arabia; hchoudhry@kau.edu.sa; 6 Biochemistry Department, Faculty of Science, King Abdulaziz University, Jeddah 21589, Saudi Arabia

**Keywords:** extracellular vesicles, exosomes, miRNAs, colorectal cancer, biomarkers, tumor microenvironment

## Abstract

Colorectal cancer (CRC) is the third most common type of cancer worldwide amongst males and females. CRC treatment is multidisciplinary, often including surgery, chemotherapy, and radiotherapy. Early diagnosis of CRC can lead to treatment initiation at an earlier stage. Blood biomarkers are currently used to detect CRC, but because of their low sensitivity and specificity, they are considered inadequate diagnostic tools and are used mainly for following up patients for recurrence. It is necessary to detect novel, noninvasive, specific, and sensitive biomarkers for the screening and diagnosis of CRC at earlier stages. The tumor microenvironment (TME) has an essential role in tumorigenesis; for example, extracellular vesicles (EVs) such as exosomes can play a crucial role in communication between cancer cells and different components of TME, thereby inducing tumor progression. The importance of miRNAs that are sorted into exosomes has recently attracted scientists’ attention. Some unique sequences of miRNAs are favorably packaged into exosomes, and it has been illustrated that particular miRNAs can be directed into exosomes by special mechanisms that occur inside the cells. This review illustrates and discusses the sorted and transported exosomal miRNAs in the CRC microenvironment and their impact on CRC progression as well as their potential use as biomarkers.

## 1. Introduction

Colorectal cancer (CRC) occurs secondary to genetic mutations in the lining mucosa of the large intestine [1]. Despite the presence of advanced screening and treatment methods for these cancers, approximately half of CRC patients die as a result of complications from the primary colonic tumor or its distant metastasis [2,3]. A variety of screening tests for CRC currently exist, but they suffer from varying sensitivity and specificity, increased cost, or invasiveness, which affects patient compliance with screening [3]. The current gold standard screening tool for CRC is colonoscopy. In addition to diagnosing CRC and localizing it within the colon, it can detect and remove the precursors of CRC, adenomatous polyps. Colonoscopy, however, is an invasive test and carries a risk of bleeding and perforation [4]. Therefore, earlier and more accurate detection of CRC is essential to decrease the risk of developing metastasis and death. There is an urgent need to find screening biomarkers that are less expensive and invasive and enable earlier detection of CRC.

Extracellular vesicles (EVs) are important molecules in the communication between cancer cells and their microenvironment [5]. Tumor cells can release different types of EVs, including secreted exosomes [6], which can participate in the progression, proliferation, and metastasis of cancer cells [7,8,9]. The exosomal cargoes, especially the microRNAs (miRNAs), play an important role in tumor progression [5,10,11,12,13]. miRNAs, small noncoding RNAs (18–25 nucleotides in length), modulate gene expression at the post-transcriptional level by triggering mRNA degradation or inhibiting the translation of mRNAs into proteins. The exosomes and their contents of miRNAs can be released from cancer cells into all body fluids (plasma, milk, urine, saliva) and can be used as early noninvasive biomarkers for cancers.

This review discusses the prevalence of CRC worldwide, the CRC microenvironment, exosome biosynthesis, and the role of exosome cargoes in tumorigenesis and progression. The impact of exosomal miRNAs on CRC tumor development and metastasis and an insight into the processes of sorting miRNAs into exosomes are also discussed.

## 2. Colorectal Cancer Incidence and Diagnosis

Colorectal cancer (CRC) is the third most common cancer in both genders [1]. There are approximately 1.4–1.93 million new cases of CRC worldwide each year [14,15].

The lack of early screening for CRC may contribute to a lower overall five-year survival rate. Untreated CRC leads to systemic metastasis, which is the main cause of death [16]. The rates of incidence and mortality of CRC in the US have declined over the last 15 years due to screening programs that rely on the early detection of the tumors and thus allow treatment at an earlier stage [17]. However, CRC case numbers are still high, while the solution is limited to surgical resection of tumors with chemotherapy [18]. The identification of CRC at an earlier stage is essential to decrease the risk of developing metastasis and death. There are many screening tools for detecting CRC; however, each diagnostic or screening method has significant limitations [19]. Moreover, it is important to identify the suitability and the efficiency of different ways of screening CRC cases while also considering the affordability of these methods for patients [20].

Endoscopy is the most widely utilized in patients as it provides a precise diagnosis of CRC. The test requires removing a tiny amount of tissue from any abnormal areas inside the rectum and colon. Endoscopy showed around 95% sensitivity and specificity for detecting polyps and CRCs. Endoscopy reduces the risk of CRC death by 65%. However, as mentioned previously, it does carry a risk of bleeding and colonic perforation [4].

Several laboratory tests have been described to help detect CRC cases, such as the fecal occult blood test (FOBT), fecal immunohistochemical test (FIT), carcinoembryonic antigen (CEA), and carbohydrate antigen 19-9 (CA19-9).

Carcinoembryonic antigen (CEA) is a serum biomarker used to evaluate the level of the CEA protein in CRC patients. CEA is usually expressed in normal intestinal epithelium cells at a low level. However, in CRC, CEA is highly elevated [21]. Despite this, it is not sensitive enough as a screening test, with only 50% of CRC patients having an elevated CEA, making it inappropriate for early-stage CRC screening [4]. It is also affected by medical conditions other than CRC. The main use for CEA remains the monitoring of CRC patients after therapy for evidence of recurrence [22]. Carbohydrate antigen 19-9 (CA19-9) is an antigen that binds monoclonal antibodies (tumor surface markers) [21]. CA19-9 is present at high levels in various gastrointestinal malignancies, including CRC. However, because of its limited sensitivity and specificity, it is regarded as an unsatisfactory diagnostic tool and is mainly used to monitor recurrence in CRC patients [23].

The fecal occult blood test (FOBT) is considered a noninvasive biomarker for CRC. This examination reveals bleeding in the gastrointestinal tract by detecting hemoglobin within the feces [4]. FOBT is an effective way to decrease CRC mortality by 13–18% [24]. FOBT alone is inadequate to establish a diagnosis of CRC, as the existence of blood in feces is not always evidence of CRC [25]. The fecal immunohistochemical test (FIT) is another widely used test for CRC screening [26]. It detects antibodies for human globin in feces. The FIT was found to be more sensitive and specific for distinguishing CRC than FOBT, as well as being a more cost-effective test [22].

Stool DNA testing is another technique for CRC screening. It works by detecting abnormal DNA associated with CRC that is shed in the stool. The stability of DNA in stool allows for easy extraction and analysis. The outcome can be used to define the immunotherapy for treating CRC patients based on the defective genes or proteins [27]. However, the cost is relatively high and the availability of this technique is usually limited [28]. Despite the efficacy of determining the genes that cause CRC, different variables may be implicated with genes.

Therefore, it is necessary to detect novel, noninvasive, specific, and sensitive biomarkers for the prognosis of CRC at earlier stages. Different miRNA levels are aberrant at different stages of CRC. As a result, these tiny RNA molecules might be helpful as clinical biomarkers for identifying cancers, selecting therapeutic interventions, and interpreting results [29].

## 3. Tumor Microenvironment and Exosomes

The tumor microenvironment (TME) is an intricate internal environmental system that is near tumor cells and plays an essential role in tumorigenesis [5]. The TME encompasses the surrounding area, including different types of cells, namely immune cells, endothelial cells, endothelial progenitor cells, epithelial cells, cancer-associated fibroblasts, normal fibroblasts, platelets, and mesenchymal stem cells (MSCs), and the extracellular matrix (ECM), which can constantly communicate with tumor cells [5,7]. The extracellular vesicles (EVs) play a particular role in this communication where they are released and passed through TME to induce tumor growth [5]. A recent study demonstrated that EVs play a critical role in transforming normal fibroblasts into cancer-associated fibroblasts (CAFs) [5]. The effect of EVs could differ based on their source. EVs derived from cancer cells and cancer stem cells can potentiate cancer progression; however, those originating from mesenchymal stem cells (MSCs) can inhibit cancer cell proliferation and metastasis [8,30]. Cancer stem cells and their EVs play key roles in the generation and development of many cancers, including CRC [31]. Several other studies have demonstrated that tumor cells can release different types of EVs, including secreted exosomes [6], which contribute to all tumorigenesis stages, including progression, proliferation, and metastasis [7,8,9]. Cancer stem cells modulate various cancer hallmarks such as proliferation of cancer cells, angiogenesis, metastasis, drug resistance, and immune dysregulation through their secreted EVs, including exosomes and MVs, in addition to some soluble factors such as chemokines, cytokines, and growth factors [32].

The exosomes are considered as the shuttle for packaging and transferring fundamental biological components between and among the cells either nearby or far away [33]. Exosomes comprise nanovesicles, around ~50–150 nm in diameter, composed of a lipid bilayer that is established from early intercellular bodies through the endosomal pathway [34,35]. Intercellular bodies contain luminal vesicles (ILV) that are introduced into multivesicular bodies (MVBs), which fuse with the plasma membrane of the cell surface to secrete exosomes [35]. In the synthesis of MVBs and ILVs, the endosomal sorting complex required for transport (ESCRT) is crucial. In addition to ESCRTs, certain proteins, such as tetraspanins (CD9, CD63, and CD81), tumor susceptibility gene 101 protein (TSG101), vacuolar protein sorting-associated protein 4 (VPS4), vesicle trafficking 1 (VTA1), and apoptosis-linked gene 2-interacting protein X (ALIX) encoded by PDCD6IP, contribute to the biogenesis process. Moreover, several mechanisms, such as heterogeneous nuclear ribonucleoprotein (hnRNP), neutral sphingomyelinase 2 (nSMase2), RNA-induced silencing complex (RISC), and miRNA post-transcriptional 3′ end modification, have been demonstrated to influence exosome synthesis [12]. Exosomes also include heat shock proteins (Hsp60, Hsp70, and Hsp90) and the molecular histocompatibility complex (MHC) [36,37]. The membranes of exosomes are rich in lipids such as lipid rafts, which include sphingolipids, sphingomyelin, glycosphingolipids, cholesterol, ceramide, and phosphatidylserine with short saturated fatty acids [38]. Moreover, exosomes interact with cells via polysialic acid on the surface of glycoconjugates [39]. Releasing exosomes is a natural process that comes from a wide variety of cells, particularly cancer cells [34]. The presence of exosomes in most vital fluids (for example, blood, urine, saliva, milk, and cerebrospinal fluid) emphasizes the significance of those exosomes [12,40,41]. Endocytosis, macropinocytosis, and phagocytosis are some of the mechanistic ways of exosome secretion [35].

Several investigations have demonstrated that exosomes are involved in various mechanisms that trigger tumor growth, enhanced migration, metastasis, escape from the immune system, and resistance to chemotherapy [30,42,43,44]. The exosomes carry the most biologically active cargos throughout the body, including metabolites, lipids, proteins, and nucleic acids (DNA, mRNA, miRNA, and other noncoding RNAs). These components have been reported to play a critical role in tumor progression [5,10,11,12,13]. According to many studies, the number of exosomes present in the blood is larger in cancer patients than in healthy controls because cancer cells release more exosomes than healthy cells [45,46]. Furthermore, the compounds found in tumor-cell-derived exosomes differ greatly from those detected in healthy cells [42]. Recent research has discovered that several proteins, long noncoding RNAs, and miRNAs in blood exosomes are considerably distinct between CRC patients and healthy controls, and these differences might be exploited for CRC diagnosis [36]. Because of these properties, exosomes are intriguing sources of potential cancer biomarkers.

## 4. MicroRNAs

**Figure 1:**
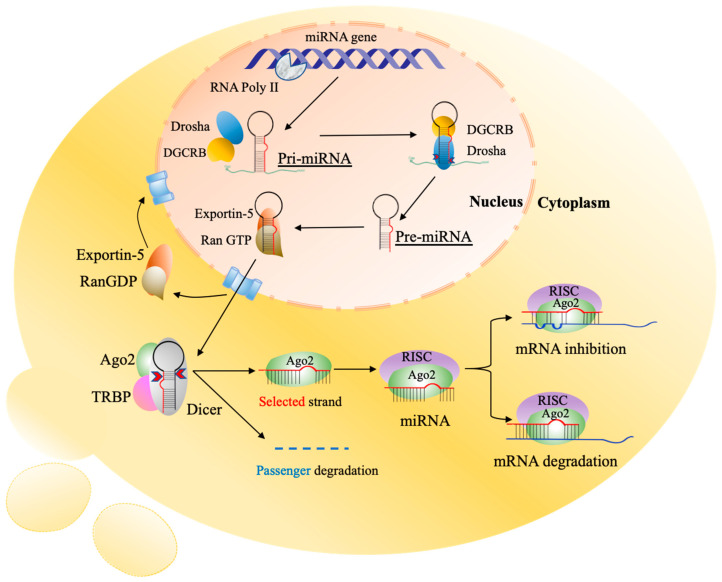
Biogenesis of miRNAs. The initiation process is started in the nucleus by RNA polymerase II which transcribes protein-coding genes into pri-miRNAs with a large cap and polyadenylation. The pri-miRNAs are processed by a complex of Drosha and RNA-binding protein DGCR8 to produce the stem-looped structures of 59–89 nucleotides (nt) known as pre-miRNAs. Then, pre-miRNAs are transported into the cytoplasm by exportin-5/Ran-GTP, and further processing occurs to generate mature miRNAs where Dicer cleaves stem-looped structures into double-strand miRNAs. One functional miRNA strand (red) is loaded with Ago2 into the RISC, and that complex, miRISC, binds to the complementary sequences of target mRNA in the 3′UTR. The result of these interactions is degradation and suppression of translation of the specific mRNA. pri-miRNAs, primary miRNAs; pre-miRNAs, processor miRNA; Dicer, RNase III nuclease; RISC, RNA-induced silencing complex; Ago2, and Argonaute 2. The image is adapted from Strubberg and Madison [18].

MicroRNAs (miRNAs) are short noncoding RNAs around 19–24 nucleotides in length that regulate transcription and post-transcriptional modifications of target genes in different cellular pathways [18]. They inhibit the translation process or degrade the transcript of a wide number of target mRNAs through binding to their 3′ untranslated region (UTR) termini, thus regulating gene expression at the protein level (Figure 1). Such a binding can affect many biological cellular processes, including proliferation and apoptosis, or occasionally initiate diseases [47,48,49].

Tumor-derived exosomal miRNAs are among the bioactive compounds that promote TME diversity, and alterations in the TME encourage tumor growth [5]. By modifying TME during tumor development, exosomes can carry miRNAs to improve cell communication and signal transduction and impact immune response [5]. For example, miR-526b and miR-655 have been shown to enhance lymphangiogenesis and angiogenesis in the TME [50], whilst miR-9 and miR-200s were found to induce tumor metastasis by stimulating NFs to transform into CAFs [51]. The ratio of miRNAs in exosomes may vary based on the type of cells, tissues, or physiological setting, but at least one of the main miRNAs is present in exosomes [36]. Exosomes operate as a shelter for protecting miRNAs and allowing them to be produced in the extracellular environment and effectively absorbed by recipient cells [52].

MSCs can secrete exosomes for different purposes such as introducing apoptosis, promoting cell cycle halt, boosting tumor cell proliferation and differentiation, and eliminating tumor-suppressor miRNAs [36]. Various tumor-suppressor miRNAs, including miR-23b, miR-921, and miR-224, are secreted selectively outside the tumor cells [53]. However, other studies have shown that miRNAs mostly released into the extracellular environment are not always the ones that are most expressed in cancer or normal cells. For example, miR-192-5p, miR-10a-5p, and miR-191-5p are typically seen in original cells, and their derived exosomes could be used as biomarkers for CRC [54,55].

**Figure 2:**
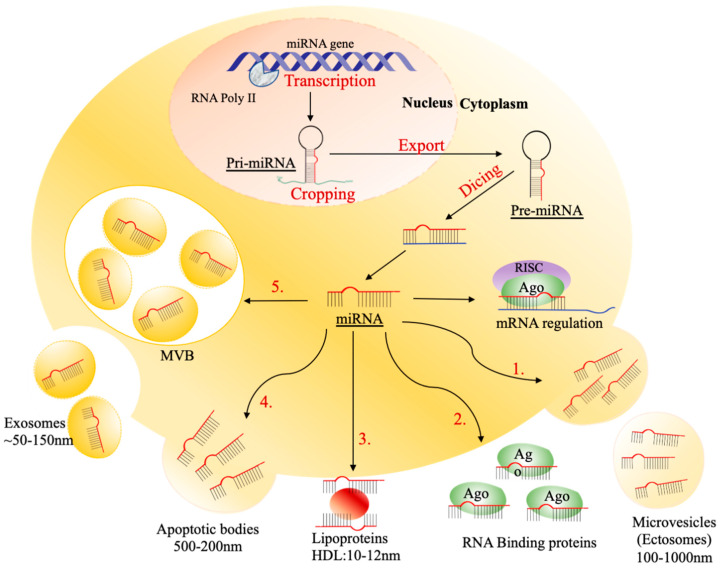
The mechanism of transporting circulating miRNAs. The process is initiated by transcription of the miRNA gene, cropping pri-miRNA, exporting pre-miRNA, and dicing to form a mature miRNA. The mature miRNA either regulates mRNA or is passaged into extracellular circulation. There are five different ways of passaging circulating miRNAs: (1) collecting in 500–200 nm of apoptotic bodies; (2) binding to Ago2 protein; (3) enveloping into 100–1000 nm of microvesicles (MVs), including ectosomes, (4) binding to 10–12 high-density lipoproteins (HDLs); (5) packaging into ~50–150 nm of exosomes.

The majority of miRNAs have been discovered inside the cells, known as intracellular miRNAs, and the group of miRNAs that can be released outside the cells are usually known as extracellular miRNAs or circulating miRNAs [56]. The importance of extracellular miRNAs is demonstrated by their abundance in biological fluids that can be packaged into the circulation form with high stability through transportation [42]. Circulating miRNA can be transported extracellularly in various ways, by collecting in apoptotic bodies; enveloping into MVs, including ectosomes; binding with RNA-binding proteins (Ago2, NPM1, or HDL); or packaging into exosomes and MVBs (Figure 2).

It has been reported that miRNAs are stably encapsulated in exosomes, which can be found in several biological fluids including seminal fluid, cerebrospinal fluid, breast milk, tears, saliva, urine [57], feces [1], and blood [58]. The bloodstream (plasma and serum) harbors abundant exosomal miRNAs that can be used as an ideal biomarker for a potential clinical diagnosis and prognosis [58] with a great capacity to resist most adverse factors such as acidic pH, basic pH, high temperature, and low temperature [59]. Furthermore, exosomal miRNAs can completely resist endogenous RNase-mediated degradation [60]. Mitchell and his colleagues, by exposing samples of exosomal miRNAs from human serum and plasma to several experimental conditions, showed that miRNAs can easily be left at room temperature for 24 h and resist thawing and freezing prosses [61]. According to these unique properties, exosomal miRNAs in the biological fluids can be used as noninvasive biomarkers with great specificity and sensitivity for the primary diagnosis of gastrointestinal malignancies [62] and notably CRC [23]. Indeed, some studies identified the downregulation of miR-24-2 in serum [63] and the upregulation of miR-129 in plasma of CRC patients [64]. Therefore, the authors suggested that these miRNAs can be used as biomarkers for detecting CRC in patients. Moreover, miRNA samples from blood stream are also valuable tools for the diagnosis of CRC [65,66].

### 4.1. Packaging and Sorting miRNAs into Exosomes

**Figure 3:**
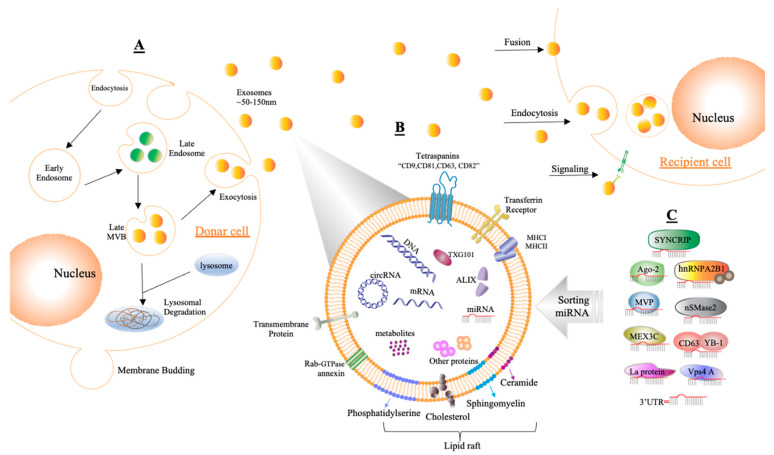
Demonstrating the composition and generation of exosomes. (**A**) Exosomes are formed by endosomal multivesicular bodies (MVBs) budding inside the cell. Some of the MVBs that develop are delivered to the membrane of the cells after becoming late MVBs. MVBs can be dissolved during fusing with the lysosome or can release exosomes into the extracellular area by fusion with the plasma membrane through the exocytosis process, and the size range of exosomes is around ~50–150 nm. Acceptor cells receive exosomes through fusion, endocytosis, and/or signaling processes to insert their content. (**B**) Exosomes are enclosed by a phospholipid bilayer that contains a variety of components on its surface, such as tetraspanins (CD9, CD81, CD63, CD82), transferrin receptors, transmembrane proteins, molecular histocompatibility complex (MHCI, MHCII), Rab-GTPase annexin, and lipid rafts, while inward components contain biological species such as RNA (circRNA, mRNA, miRNA), proteins, DNA, and metabolites. In addition, tumor susceptibility gene 101 (TSG101) and apoptosis-linked gene 2-interacting protein X (ALIX) can be used as markers for exosomes. (**C**) Sorting miRNAs into exosomes can be regulated via different binding processes such as those of synaptotagmin-binding cytoplasmic RNA-interacting protein (SYNCRIP), sumoylated hnRNPA2B, Argonaute protein (Ago2), neutral sphingomyelinase 2 (nSMase2), major vault protein (MVP), CD63 with Y-box protein I (YBP1), Mex-3 RNA-binding family member C (MEX3C), protein 4A (Vps4A), lupus La protein (La protein), or the 3′ end of miRNA (3′UTR).

Several studies reported that exosomes have been used as a shuttle to transfer miRNAs between cells, which influences the biological processes of the acceptor cells. In 2018, the recorded number of mature microRNAs encoded by the human genome was around 2600 (miRBase v.22) based on the result of Xiaoyi and his group, who detected 593 exosomal miRNAs in human biopsy fluid by sequencing [67]. The processes of sorting miRNAs into exosomes have not been clearly explained; however, recent reports suggested some mechanisms that could be related to packaging miRNAs in exosomes [5]. Exosomes utilize two types of miRNA sorting mechanisms (selective and nonselective), presumably based on exosome source [68] (Figure 3).

The Argonaute protein (Ago2) with RNA-induced silencing complex (RISC) is involved as a functional holder of miRNAs that is specifically related to sorting some miRNAs, such as miR-142-3p, miR-150 and miR-451, into exosomes of let-7a, miR-100, and miR-320a in human embryonic kidney HEK293T cells [69,70,71]. Moreover, three members of the family of heterogeneous nuclear ribonucleoproteins, A1, A2/B1, and Q (hnRNPA1, -A2/B1, and -Q), have been associated with loading miRNAs into exosomes [69]. hnRNPA1 is an RNA-binding protein (RBP) that was identified to interact with miRNA sequences such as miR-522, leading to its loading into the exosome [72]. In addition, miR-582-5p was packed into exosomes derived from human colon cancer SW620 cells [73]. A recent report by Xu et al. demonstrated that the motif sequence GCAG in the RUN2-1 transcript is responsible for enriching miR-1246 in the exosome of human cancer cells [74]. hnRNPA2B1 is a sumoylated RNA-interacting protein showing “EXO-motifs” that are responsible for recognizing the sequence of the specific motif of miRNAs and regulating their localization into exosomes [69,75]. The composite of miRNA–hnRNPA2B1 was identified in the exosomes of human cerebrospinal fluid, which supports the association of hnRNPA2B1 in directing miRNA into exosomes [76]. Around 30 miRNAs were sorted into exosomes by identifying GGAG motifs by hnRNPA2B1 [73]. Moreover, two miRNAs, namely miR-198 and miR-601, were identified at the recognition motif site GGAG/UGCA at the 3’ end of miRNAs interacting with hnRNPA2/B1 for transportation into exosomes [75].

hnRNPQ, known as SYNCRIP, is a conserved RNA-interacting protein that can distinguish several sequences of exosomal miRNAs harboring the GGCU motif [77]. In addition, the GCUG motif at the 3’ end of miRNAs is recognized by hnRNPQ, and hnRNPQ packs miRNAs into exosomes [77]. For instance, the addition of GCUG motif sequence into miR29a-1 (nontarget miRNA) resulted in the successful loading into exosomes by hnRNPQ detection [78]. The hnRNPA2B1 can bind miR-1246 via the GGAG motif to regulate its loading into exosomes [78]. Furthermore, sumoylation can regulate the binding of hnRNPA2B1 to miRNAs [75]. Sumoylated hnRNPA2B1 was found to be three times more abundant in mutant p53 CRC cells than in normal cells, indicating that modifications in this pathway are implicated in exosomal miR-1246 carcinogenic characteristics [79].

Neutral sphingomyelinase 2 (nSMase2), an enzyme that contributes to exosomal biogenesis by the formation of ceramide (a part of exosomal membranes), is involved in the secretion process of exosomal miRNA [80]. The study of Kosaka et al. demonstrated that a high level of nSMase2 expression increases miRNA loading into exosomes, while the inhibition of nSMase2 prevents exosomal development and the loading of some miRNAs [81]. The authors also revealed that miR-146a and miR-16 are enriched in exosomes because of the high level of nSMase2 expression, while the source cells conserve the same number of miRNAs [82]. The authors suggested that ceramide can also be responsible for sorting several miRNAs, while the inhibition of ceramide by GW4869 influences the expression of exosomal miRNAs such as miR-451a [81]. Further, nSMase2 was shown to increase angiogenesis and metastasis within the TME by regulating tumor-derived exosomal miRNAs [81].

Protein 4A (Vps4A), associated with vacuolar protein sorting, is a membrane protein that is involved in the regulation of some exosomal miRNAs in human hepatoma cells (Wei et al., 2015). miR-132-3p, miR-320a, and miR-193a-3p were enriched in exosomes according to high Vps4A expression, while low exosomal miR-92a and miR-150A levels were indicated as the result of Vps4A suppression [83]. Another RNA-binding protein, namely Y-box protein I (YBP1), a transport protein, is released from the cells by exosomes. YBP1 was found to regulate miRNA sorting by interacting with CD63 to recognize a specific sequence of miRNAs, such as miR-223, and direct it into the exosome. In addition, the YBP1 mutant was recognized to reduce the number of sorting miRNAs such as miR-223 and miR-144 in exosomes [84,85].

Mex-3 RNA-binding family member C (MEX3C) is an RNA-binding E3 ubiquitin ligase that is required for the mRNA degradation process [86]. Recent data indicated that MEX3C may also contribute to the sorting of miRNA into exosomes, and the Ag2 protein was associated with this process. In addition, there was no match sequence between miR-451a and MEX3C, but the complex of Ago2–MEX3C may promote the binding of miRNAs and drive them into exosomes [86]. Lue et al. (2017) also examined the role of MEX3C in enriching exosomal miRNA by applying siRNA to inhibit MEX3C, hence reducing the expression level of exosomal miR-451a. Another study proved the role of other types of RNA-binding protein, such as lupus La protein, which acts as a selective carrier for transporting specific miRNAs into exosomes to promote cancer metastasis [87]. Lupus La protein can recognize the two motifs of miR-122 and localize them into exosomes [68].

Major vault protein (MVP) interacts with miR-193a and promotes cancer development. Exosomal miR-193a is abundantly expressed in CRC patients. Inhibition of MVP can result in the accumulation of miR-193a in the cell rather than in the exosomes, thereby suppressing cancer development [88]. Lipid rafts also significantly contribute to exosome formation and provide exosomes with more stiffness than plasma membranes [38]. A lipid raft is another ESCRT that can associate with the exosome packaging process based on the binding to RNA sequences with 10 specific motifs (UGCC GGCG, GGAC, two UCCG motifs, two UGAC motifs, and three GCCG motifs) [89]. In a recent study, researchers discovered four motifs that appear to be the most common in both exosomal protumoral miRNAs and lipid raft RNA-binding motifs: UCCC, UUGU, CUCC, and CCCU [39]. Another mechanism that is thought to load miRNAs into exosomes involves the 3′ end of miRNA. The study of isolated exosomal miRNAs from both human urine and B cells demonstrated that the uridine-rich motifs at the 3′ ends of exosomal miRNAs are responsible for loading into exosomes, although the source cells had adenine-rich motifs at the 3′ ends [90]. It was shown that miRNAs may preferentially bundle RNA payloads into exosomes; for example, upregulation of miR-1289 was demonstrated to promote the packaging of GalR3 mRNA into exosomes [91].

In general, sorting miRNAs into exosomes is a highly selective process that can be dominated by the target sequences of endogenous RNA and miRNAs, many of the latter being tumor-suppressive (Table 1) [69,92]. Tumor cells can also change the level of loading of miRNAs into exosomes by regulating the signaling pathway of sorting exosomal miRNA. KRAS mutation in CRC was shown to affect the loading of miRNA into exosomes with the nSMase2 process, and miR-100 was enriched and packaged into exosomes. However, in normal cells, KRAS delivers a great amount of miR-10 into exosomes [93]. As a result, cancer cells may employ many EVs at the same time via independent sorting processes, allowing varied activities in normal or malignant cases [68].

### 4.2. The Role of Exosomal miRNAs in CRC Progression and Drug Resistance

Exosomes can contribute to CRC development and promote CRC progression. Such CRC cells release exosomal miRNAs within the TME or distant organs through the blood to transport information to the acceptor cells, thus facilitating CRC development, metastasis, and chemoresistance [42]. Cancer drug resistance is either fundamental or vested. The former exists in the body before treatment, while the latter occurs during the treatment [111]. Resistance to anticancer drugs has a substantial impact on the efficacy of chemotherapeutic and molecular targeted therapies, potentially leading to a poor prognosis and cancer recurrence [112]. CRC cells can resist treatment through a variety of mechanisms, including drug efflux, drug target mutations, drug metabolism modifications, DNA damage repair, energy programming modifications, cancer stem cells, and epigenetic modifications. The exosomal miRNAs play a critical role in this therapeutic resistance. In the incidence and development of chemoresistance, exosomal miRNAs have two strategies. First, miRNAs can either support tumor cells becoming more chemoresponsive or support tumor cells becoming resistant to chemotherapy [112]. The second malfunction of pharmacokinetic variables, such as absorption, distribution, metabolism, and elimination (ADME), is significantly associated with drug resistance. The latter is aided by exosomal miRNA, which interferes with drug efflux and metabolism.

The expression of ATP-binding cassette (ABC) transporters is regulated by tumor-derived exosomal miRNA cargos, which improve drug resistance in cancer cells. ATP-binding cassette sub-family B member 1 (ABCB1) is an ABC transporter regulator of drug efflux that is found in microvesicles and exosomes released by chemoresistant cells [113]. A recent study demonstrated that downregulation of exosomal miR-128-3p has a critical role in chemoresistant CRC, especially for oxaliplatin drugs [114]. Exosomal miR-128-3p is a tumor-suppressor miRNA that regulates ABCC5, which is a part of the ABC transporter family also known as MRP5. Highly expressed miR-128-3p inhibits oxaliplatin efflux by suppressing MRP5 expression. Furthermore, reduced oxaliplatin efflux may lead to increased drug intracellular distribution, resulting in damaging DNA that eventually kills tumor cells [114]. Additionally, exosomal miR-128-3p controls the expression of E-cadherin in CRC cells during the binding of the 3’UTR in the *Bmi1* gene. However, the low expression of miR-128-3p increases *Bmi1* expression, which induces epithelial–mesenchymal transition (EMT), making CRC cells more resistant to oxaliplatin. Thus, the combination of miR-128-3p overexpression and oxaliplatin drug can be used to reduce the formation of resistant CRC cells more efficiently than oxaliplatin alone [114].

Tumor cells can repair DNA damage and manage cell cycle arrest, thus allowing the cells to continue to grow and proliferate. To suppress cell growth, genotoxic substances are used to damage DNA or prevent the synthesis of new DNA, which is the target of these anticancer drugs. Direct genotoxic drugs, such as cisplatin, can inflict direct damage, whereas indirect drugs, such as topoisomerase inhibitors, can inflict indirect damage [115]. Moreover, the DNA damage response (DDR) involves DNA damage repair, in addition to cell death [116]. Because of the extensive usage of genotoxic substances, cancer cells can exploit DNA damage repair as a resistance strategy [117]. In CRC, exosomal let-7g and miR-181b were determined to regulate various important genes, such as *RAS*, *cyclin D*, *C-myc*, *E2F,* and cytochrome C, that are responsible for cell signaling, cell cycle control, and chemosensitivity. Both let-7g and miR-181b are strongly associated with CRC patients’ response to 5-fluorouracil-based antimetabolite S-1 chemotherapy [118].

Exosomal miR-1246 overexpression in CRC inhibits cell proliferation, invasion, migration, and apoptosis by suppressing the production of CCNG2 (cyclin G2 or CycG2) [119]. The expression of miR-1246 was found to be correlated with chemoresistance and cancer stem cell-like features through CCNG2 inhibition, suggesting the poor prognosis of patients’ pancreatic tumors [120]. These findings showed that overexpression of miR-1246 might be a biological process underlying CCNG2 regression in pancreatic tumor cells [120]. Another study on exosomal miR-1246 found that downregulation of CCNG2 is associated with high expression of exosomal miR-1246 in breast cancer, which can increase tumor growth, invasion, and chemotherapy resistance [121]. However, it is important to check whether these findings are also applicable in CRC cells. To detect whether miR-1246 impacts the chemoresistance of CRC cells and stemness of CRC stem cells, more research is needed.

Energy source modification has long been recognized as a characteristic feature of tumor cells [122]. Tumor cells must modify their metabolism to meet the rising energy requirement in terms of maintaining survival, reproduction, and spread [123]. To sustain the physiological activities of the cells, metabolic processes such as glycolysis and mitochondrial oxidative phosphorylation (OXPHOS) work together to create adenosine triphosphate (ATP). The increase in glycolysis is a typical feature of both primary and metastatic tumors [124], and anoxic conditions are ideal for glycolysis. Nevertheless, tumor cells perform aerobic glycolysis by altering glucose metabolism even under aerobic settings, and glycolysis in the TME is wildly prevalent. However, increased lactate synthesis is a significant process in tumor cells that causes an acidic TME, and a decrease in extracellular pH can cause a decrease in cytotoxic T-cell activity. The acidic TME contributes significantly to immunologic escape, thus allowing tumor cells to gain considerable survival benefits that support tumor dissemination, migration, and drug resistance [112,123,125].

miRs may target both tumor suppressors and oncogenes, giving a very complicated phenotypic consequence. For example, miR-140 expression inhibits cell growth of CRC and causes cell cycle arrest [126], while overexpression of miR-140 can cause resistance to TS Tomudex (TDX) and methotrexate (MTX) therapies in CRC [126]. CRC stem cells were activated to respond to 5-FU therapy by inhibiting miR-140 activity with LNA oligonucleotides of anti-miR-140 [126]. Further investigations are required to indicate whether high levels of miR-215 and miR-140 are found in CRC stem-like cells that are known to be resistant to 5-FU therapy.

## 5. Conclusions

The detection of exosomes and their cargoes, especially miRNAs, is fundamental in explaining the mechanisms of tumor–microenvironment crosstalking and modulation of migration- and invasion-related genes. Considering their impact on CRC pathogenesis, exosomes and their miRNAs can act as therapeutic targets, either by repressing exosomes’ biogenesis, secretion, and uptake or by inhibiting oncogenic exosomal miRNAs that can participate in tumor progression. On the other hand, some exosomal miRNAs are beneficial and have a tumor-suppressive role; they can be used as therapeutic agents. Exosomal miRNAs can be also considered promising sources of inexpensive, noninvasive, and more accurate biomarkers for the diagnosis and prognosis of CRC. However, further work is needed to surmount the technical challenges restricting the clinical application of exosomes and their miRNAs as early diagnostic biomarkers for CRC.

## Figures and Tables

**Table 1:** miRNAs sorted into exosomes with known functions in CRC.

miRNA	Function
miR-671-5p [94]	Oncogenic miR that is overexpressed in the large intestine of CRC patients and colorectal cancer cell lines. Its expression is associated with metastasis, proliferation, migration, and invasion of CRC cells.
miR-193b [95]	Tumor-suppressive miR that is downregulated in the serum of CRC patients. Its low levels are correlated with TNM stage and metastasis in CRC patients.
miR-1224-5p [96]	Tumor-suppressive miR that is reduced in CRC tissues and cell lines mainly due to the hypoxic microenvironment. It prevents the epithelial–mesenchymal transition (EMT), invasion, and migration of CRC cells by directly interfering with the SP1-mediated NF-κB pathway.
miR-125b-1 [97]	Tumor-suppressive miR that is downregulated in early CRC cell lines. Its low levels induce metastasis by increasing the expression of the *XIAP* gene.
miR-125a-3p [98]	Tumor-suppressive miR suppressing fucosyltransferase (FUT)5 and FUT6 to regulate the PI3K/Akt signaling pathway, subsequently inhibiting the proliferation, migration, invasion, and angiogenesis of CRC cells. It also inhibits CRC development by directly targeting the angiogenesis-related gene VEGFA and the antiapoptotic gene Bcl-2.
miR-483-5p [99]	Tumor-suppressive miR that inhibits CRC cell proliferation and metastasis, possibly through inhibiting tumor necrosis factor-receptor associated factor (TRAF), which plays critical roles in immune cell signaling).
miR-188-5p [100]	Oncogenic miR that is overexpressed in CRC tissue and cell lines. Its higher expression is accompanied by tumor cell proliferation, invasion, and metastasis through inhibition of FOXL1/Wnt signaling.
miR-765 [101]	Tumor-suppressive miR that inhibits proliferation, migration, and invasion of CRC cells by targeting patatin-like protein 2 (PLP2).
miR-638 [102]	Tumor-suppressive miR that is downregulated in the serum and within exosomes of CRC patients and cell lines. It represses CRC cell viability and migration and modulates the cell cycle by inhibiting tetraspanin 1 (TSPAN1). A reduction in miR-638 is associated with poor overall survival.
miR-1246 [79]	Oncogenic miR that is involved in tumor progression and metastasis. miR-1246 targets normal p53; however, miR-1246 was found to be overexpressed in mutant p53 tumor-derived exosomes.
miR-654-5p [103]	The abundant expression of miR-654-5p is correlated with colon cancer development, metastasis, and a low survival rate of CRC patients.
miR-17 [104]	Overexpression of miR-17 can increase cell proliferation and liver metastases in CRC. It is also associated with the progression of colorectal adenoma to adenocarcinoma in CRC patients.
miR-198 [105]	Tumor-suppressive miR that hinders CRC cell viability, triggers death, and inhibits metastasis.
miR-601 and miR-760 [54]	Their expression was reduced in the serum of CRC patients, and they could be used as predictors of advanced CRC.
miR-493-5p [106]	Tumor-suppressive miR that inhibits CRC progression by targeting the PI3K–Akt–FoxO3a signaling pathway.
miR-223-3p [107]	Oncogenic miR that is upregulated in CRC tissues and associated with the proliferation and metastasis of CRC cells.
miR-320 [108]	Tumor-suppressive miR that is downregulated in CRC tissues and cell lines. Its upregulation is associated with the inhibition of CRC cell proliferation and metastasis. Its downstream targets are *FOXQ1* and *SOX4* genes.
miR-486-5p [109]	Tumor-suppressive miR that is downregulated in CRC tissues. It is a negative regulator of pleiomorphic adenoma gene-like 2 (PLAGL2), a transcription factor for β-catenin and insulin-like growth factor 2 (IGF2) with roles in promoting proliferation, cell survival, and metastasis, as well as decreasing E-cadherin and increasing N-cadherin expression.
miR-150 [110]	Tumor-suppressive miR that is downregulated in serum exosomes of CRC patients; however, this expression was increased in postoperative samples. The downregulated expression was associated with higher tumor progression, metastasis, and poor survival rate.
miR-100 [97]	When the expression of this tumor-suppressive miR decreased, CRC growth and metastasis increased. The mechanism involves the induction of downstream targets *mTOR*, *IGF1R*, *Fas,* and *XIAP*.
miR-92a-3p [98]	Exosomal miR-92a-3p facilitates tumor angiogenesis by inducing partial EMT in endothelial cells and through the downregulation of Dkk-3 and claudin-11. Exosomes derived from colon cancer cells and plasma derived from murine xenograft models were enriched with miR-92a-3p, and it has been found to stimulate tube formation in human umbilical vein endothelial cells upon transfer.
miR-193a [88]	The expression of this tumor-suppressive miR is abundantly increased in the exosomes of metastatic CRC cell lines and plasma of CRC patients with liver metastasis. Its upregulated expression is associated with a cell cycle arrest in the G1 phase followed by the hindering of CRC cell proliferation through the inhibition of Caprin1, followed by CCND2 and c-MYC. The loss of major vault protein (MVP) caused the upregulation of miR-193a in cells rather than exosomes.

## References

[1] Ding L., Lan Z., Xiong X., Ao H., Feng Y., Gu H., Yu M., Cui Q. (2018). The Dual Role of MicroRNAs in Colorectal Cancer Progression. Int. J. Mol. Sci..

[2] Augestad K., Bakaki P., Rose J., Crawshaw B., Lindsetmo R., Dørum L., Koroukian S., Delaney C. (2015). Metastatic spread pattern after curative colorectal cancer surgery. A retrospective, longitudinal analysis. Cancer Epidemiol..

[3] Toiyama Y., Okugawa Y., Fleshman J., Boland C.R., Goel A. (2018). MicroRNAs as potential liquid biopsy biomarkers in colorectal cancer: A systematic review. Biochim. Biophys. Acta Rev. Cancer.

[4] Swiderska M., Choromańska B., Dąbrowska E., Konarzewska-Duchnowska E., Choromańska K., Szczurko G., Myśliwiec P., Dadan J., Ladny J.R., Zwierz K. (2014). The diagnostics of colorectal cancer. Contemp. Oncol..

[5] Tan S., Xia L., Yi P., Han Y., Tang L., Pan Q., Tian Y., Rao S., Oyang L., Liang J. (2020). Exosomal miRNAs in tumor microenvironment. J. Exp. Clin. Cancer Res..

[6] Huang T., Deng C.-X. (2019). Current progresses of exosomes as cancer diagnostic and prognostic biomarkers. Int. J. Biol. Sci..

[7] Ingenito F., Roscigno G., Affinito A., Nuzzo S., Scognamiglio I., Quintavalle C., Condorelli G. (2019). The role of exo-miRNAs in cancer: A focus on therapeutic and diagnostic applications. Int. J. Mol. Sci..

[8] Alzahrani F.A., El-Magd M.A., Abdelfattah-Hassan A., Saleh A.A., Saadeldin I.M., El-Shetry E.S., Badawy A.A., Alkarim S. (2018). Potential Effect of Exosomes Derived from Cancer Stem Cells and MSCs on Progression of DEN-Induced HCC in Rats. Stem Cells Int..

[9] Badawy A.A., El-Magd M.A., AlSadrah S.A. (2018). Therapeutic Effect of Camel Milk and Its Exosomes on MCF7 Cells In Vitro and In Vivo. Integr. Cancer Ther..

[10] Yang E., Wang X., Gong Z., Yu M., Wu H., Zhang D. (2020). Exosome-mediated metabolic reprogramming: The emerging role in tumor microenvironment remodeling and its influence on cancer progression. Signal Transduct. Target. Ther..

[11] Vaidya M., Sugaya K. (2020). Differential sequences and single nucleotide polymorphism of exosomal SOX2 DNA in cancer. PLoS ONE.

[12] Dai J., Su Y., Zhong S., Cong L., Liu B., Yang J., Tao Y., He Z., Chen C., Jiang Y. (2020). Exosomes: Key players in cancer and potential therapeutic strategy. Signal Transduct. Target. Ther..

[13] Chen C., Luo Y., He W., Zhao Y., Kong Y., Liu H., Zhong G., Li Y., Li J., Huang J. (2020). Exosomal long noncoding RNA LNMAT2 promotes lymphatic metastasis in bladder cancer. J. Clin. Investig..

[14] WHO (2020). http://www.who.int/cancer/en/.

[15] Zhu M., Huang Z., Zhu D., Zhou X., Shan X., Qi L.-W., Wu L., Cheng W., Zhu J., Zhang L. (2017). A panel of microRNA signature in serum for colorectal cancer diagnosis. Oncotarget.

[16] Wang L.G., Gu J. (2012). Serum microRNA-29a is a promising novel marker for early detection of colorectal liver metastasis. Cancer Epidemiol..

[17] Alomar S.Y., Mansour L., Abuderman A., Alkhuriji A., Arafah M., Alwasel S., Harrath A.H., Almutairi M., Trayhyrn P., Dar J.A. (2016). β-Catenin accumulation and S33F mutation of CTNNB1 gene in colorectal cancer in Saudi Arabia. Pol. J. Pathol..

[18] Strubberg A.M., Madison B.B. (2017). MicroRNAs in the etiology of colorectal cancer: Pathways and clinical implications. Dis. Models Mech..

[19] Zanutto S., Ciniselli C.M., Belfiore A., Lecchi M., Masci E., Delconte G., Primignani M., Tosetti G., Dal Fante M., Fazzini L. (2019). Plasma miRNA-based signatures in CRC screening programs. Int. J. Cancer.

[20] Alsanea N., Almadi M.A., Abduljabbar A.S., Alhomoud S., Alshaban T.A., Alsuhaibani A., Alzahrani A., Batwa F., Hassan A.-H., Hibbert D. (2015). National Guidelines for Colorectal Cancer Screening in Saudi Arabia with strength of recommendations and quality of evidence: Tripartite Task Force from Saudi Society of Colon & Rectal Surgery, Saudi Gastroenterology Association and Saudi Oncology Society. Ann. Saudi Med..

[21] Kim N.H., Lee M.Y., Park J.H., Park D.I., Sohn C.I., Choi K., Jung Y.S. (2017). Serum CEA and CA 19-9 levels are associated with the presence and severity of colorectal neoplasia. Yonsei Med. J..

[22] Vega P., Valentín F., Cubiella J. (2015). Colorectal cancer diagnosis: Pitfalls and opportunities. World J. Gastrointest. Oncol..

[23] Chen B., Xia Z., Deng Y.N., Yang Y., Zhang P., Zhu H., Xu N., Liang S. (2019). Emerging microRNA biomarkers for colorectal cancer diagnosis and prognosis. Open Biol..

[24] Hassan C., Rossi P.G., Camilloni L., Rex D., Jimenez-Cendales B., Ferroni E., Borgia P., Zullo A., Guasticchi G., Group H. (2012). Meta-analysis: Adherence to colorectal cancer screening and the detection rate for advanced neoplasia, according to the type of screening test. Aliment. Pharmacol. Ther..

[25] Levin B., Lieberman D.A., McFarland B., Smith R.A., Brooks D., Andrews K.S., Dash C., Giardiello F.M., Glick S., Levin T.R. (2008). Screening and surveillance for the early detection of colorectal cancer and adenomatous polyps, 2008: A joint guideline from the American Cancer Society, the US Multi-Society Task Force on Colorectal Cancer, and the American College of Radiology. CA Cancer J. Clin..

[26] Mousavinezhad M., Majdzadeh R., Sari A.A., Delavari A., Mohtasham F. (2016). The effectiveness of FOBT vs. FIT: A meta-analysis on colorectal cancer screening test. Med. J. Islamic Repub. Iran.

[27] Obuch J.C., Ahnen D.J. (2016). Colorectal cancer: Genetics is changing everything. Gastroenterol. Clin..

[28] LeGolvan M.P., Taliano R.J., Resnick M.B. (2012). Application of molecular techniques in the diagnosis, prognosis and management of patients with colorectal cancer: A practical approach. Hum. Pathol..

[29] Wang H., Peng R., Wang J., Qin Z., Xue L. (2018). Circulating microRNAs as potential cancer biomarkers: The advantage and disadvantage. Clin. Epigenet..

[30] Zahran R., Ghozy A., Elkholy S.S., El-Taweel F., El-Magd M.A. (2020). Combination therapy with melatonin, stem cells and extracellular vesicles is effective in limiting renal ischemia–reperfusion injury in a rat model. Int. J. Urol..

[31] Su C., Zhang J., Yarden Y., Fu L. (2021). The key roles of cancer stem cell-derived extracellular vesicles. Signal Transduct. Target. Ther..

[32] López de Andrés J., Griñán-Lisón C., Jiménez G., Marchal J.A. (2020). Cancer stem cell secretome in the tumor microenvironment: A key point for an effective personalized cancer treatment. J. Hematol. Oncol..

[33] Martellucci S., Orefice N.S., Angelucci A., Luce A., Caraglia M., Zappavigna S. (2020). Extracellular Vesicles: New Endogenous Shuttles for miRNAs in Cancer Diagnosis and Therapy?. Int. J. Mol. Sci..

[34] Doyle L.M., Wang M.Z. (2019). Overview of extracellular vesicles, their origin, composition, purpose, and methods for exosome isolation and analysis. Cells.

[35] Hessvik N.P., Llorente A. (2018). Current knowledge on exosome biogenesis and release. Cell. Mol. Life Sci..

[36] Vautrot V., Chanteloup G., Elmallah M., Cordonnier M., Aubin F., Garrido C., Gobbo J. (2019). Exosomal miRNA: Small molecules, big impact in colorectal cancer. J. Oncol..

[37] Lv L.-H., Wan Y.-L., Lin Y., Zhang W., Yang M., Li G.-L., Lin H.-M., Shang C.-Z., Chen Y.-J., Min J. (2012). Anticancer drugs cause release of exosomes with heat shock proteins from human hepatocellular carcinoma cells that elicit effective natural killer cell antitumor responses in vitro. J. Biol. Chem..

[38] Kalra H., Drummen G.P., Mathivanan S. (2016). Focus on extracellular vesicles: Introducing the next small big thing. Int. J. Mol. Sci..

[39] Janas T., Janas P., Sapoń K., Janas T. (2020). Binding of RNA Aptamers to Membrane Lipid Rafts: Implications for Exosomal miRNAs Transfer from Cancer to Immune Cells. Int. J. Mol. Sci..

[40] Badawy A.A., Othman R.Q.A., El-Magd M.A. (2021). Effect of combined therapy with camel milk-derived exosomes, tamoxifen, and hesperidin on breast cancer. Mol. Cell. Toxicol..

[41] Ibrahim H.M., Mohammed-Geba K., Tawfic A.A., El-Magd M.A. (2019). Camel milk exosomes modulate cyclophosphamide-induced oxidative stress and immuno-toxicity in rats. Food Funct..

[42] Xiao Y., Zhong J., Zhong B., Huang J., Jiang L., Jiang Y., Yuan J., Sun J., Dai L., Yang C. (2020). Exosomes as potential sources of biomarkers in colorectal cancer. Cancer Lett..

[43] Gerloff D., Lützkendorf J., Moritz R.K., Wersig T., Mäder K., Müller L.P., Sunderkötter C. (2020). Melanoma-derived exosomal miR-125b-5p educates tumor associated macrophages (TAMs) by targeting lysosomal acid lipase A (LIPA). Cancers.

[44] Daassi D., Mahoney K.M., Freeman G.J. (2020). The importance of exosomal PDL1 in tumour immune evasion. Nat. Rev. Immunol..

[45] Matsumura T., Sugimachi K., Iinuma H., Takahashi Y., Kurashige J., Sawada G., Ueda M., Uchi R., Ueo H., Takano Y. (2015). Exosomal microRNA in serum is a novel biomarker of recurrence in human colorectal cancer. Br. J. Cancer.

[46] Ogata-Kawata H., Izumiya M., Kurioka D., Honma Y., Yamada Y., Furuta K., Gunji T., Ohta H., Okamoto H., Sonoda H. (2014). Circulating exosomal microRNAs as biomarkers of colon cancer. PLoS ONE.

[47] Francavilla A., Turoczi S., Tarallo S., Vodicka P., Pardini B., Naccarati A. (2020). Exosomal microRNAs and other non-coding RNAs as colorectal cancer biomarkers: A review. Mutagenesis.

[48] Plotnikova O., Baranova A., Skoblov M. (2019). Comprehensive analysis of human microRNA–mRNA interactome. Front. Genet..

[49] Badawy A.A., El-Magd M.A., AlSadrah S.A., Alruwaili M.M. (2020). Altered expression of some miRNAs and their target genes following mesenchymal stem cell treatment in busulfan-induced azoospermic rats. Gene.

[50] Hunter S., Nault B., Ugwuagbo K.C., Maiti S., Majumder M. (2019). Mir526b and Mir655 promote tumour associated angiogenesis and lymphangiogenesis in breast cancer. Cancers.

[51] Baroni S., Romero-Cordoba S., Plantamura I., Dugo M., D’ippolito E., Cataldo A., Cosentino G., Angeloni V., Rossini A., Daidone M. (2016). Exosome-mediated delivery of miR-9 induces cancer-associated fibroblast-like properties in human breast fibroblasts. Cell Death Dis..

[52] Dilsiz N. (2020). Role of exosomes and exosomal microRNAs in cancer. Future Sci. OA.

[53] Ostenfeld M.S., Jeppesen D.K., Laurberg J.R., Boysen A.T., Bramsen J.B., Primdal-Bengtson B., Hendrix A., Lamy P., Dagnaes-Hansen F., Rasmussen M.H. (2014). Cellular disposal of miR23b by RAB27-dependent exosome release is linked to acquisition of metastatic properties. Cancer Res..

[54] Wang Q., Huang Z., Ni S., Xiao X., Xu Q., Wang L., Huang D., Tan C., Sheng W., Du X. (2012). Plasma miR-601 and miR-760 are novel biomarkers for the early detection of colorectal cancer. PLoS ONE.

[55] Xi Y., Formentini A., Chien M., Weir D.B., Russo J.J., Ju J., Kornmann M., Ju J. (2006). Prognostic values of microRNAs in colorectal cancer. Biomark. Insights.

[56] Sohel M.H. (2016). Extracellular/circulating microRNAs: Release mechanisms, functions and challenges. Achiev. Life Sci..

[57] Nedaeinia R., Manian M., Jazayeri M., Ranjbar M., Salehi R., Sharifi M., Mohaghegh F., Goli M., Jahednia S., Avan A. (2017). Circulating exosomes and exosomal microRNAs as biomarkers in gastrointestinal cancer. Cancer Gene Ther..

[58] Kumar S., Vijayan M., Bhatti J., Reddy P.H. (2017). MicroRNAs as peripheral biomarkers in aging and age-related diseases. Prog. Mol. Biol. Transl. Sci..

[59] Igaz P. (2015). Circulating Micrornas in Disease Diagnostics and Their Potential Biological Relevance.

[60] Koga Y., Yasunaga M., Moriya Y., Akasu T., Fujita S., Yamamoto S., Matsumura Y. (2011). Exosome can prevent RNase from degrading microRNA in feces. J. Gastrointest. Oncol..

[61] Mitchell P.S., Parkin R.K., Kroh E.M., Fritz B.R., Wyman S.K., Pogosova-Agadjanyan E.L., Peterson A., Noteboom J., O’Briant K.C., Allen A. (2008). Circulating microRNAs as stable blood-based markers for cancer detection. Proc. Natl. Acad. Sci. USA.

[62] Shigeyasu K., Toden S., Zumwalt T.J., Okugawa Y., Goel A. (2017). Emerging role of microRNAs as liquid biopsy biomarkers in gastrointestinal cancers. Clin. Cancer Res..

[63] He H., Wang N., Yi X., Tang C., Wang D. (2018). Low-level serum miR-24-2 is associated with the progression of colorectal cancer. Cancer Biomark..

[64] Ya G., Wang H., Ma Y., Hu A., Ma Y., Hu J., Yu Y. (2017). Serum miR-129 functions as a biomarker for colorectal cancer by targeting estrogen receptor (ER) β. Die Pharm. Int. J. Pharm. Sci..

[65] Hur K., Toiyama Y., Okugawa Y., Ide S., Imaoka H., Boland C.R., Goel A. (2017). Circulating microRNA-203 predicts prognosis and metastasis in human colorectal cancer. Gut.

[66] Ng L., Wan T.M.-H., Man J.H.-W., Chow A.K.-M., Iyer D., Chen G., Yau T.C.-C., Lo O.S.-H., Foo D.C.-C., Poon J.T.-C. (2017). Identification of serum miR-139-3p as a non-invasive biomarker for colorectal cancer. Oncotarget.

[67] Huang X., Yuan T., Tschannen M., Sun Z., Jacob H., Du M., Liang M., Dittmar R.L., Liu Y., Liang M. (2013). Characterization of human plasma-derived exosomal RNAs by deep sequencing. BMC Genom..

[68] Temoche-Diaz M.M., Shurtleff M.J., Nottingham R.M., Yao J., Fadadu R.P., Lambowitz A.M., Schekman R. (2019). Distinct mechanisms of microRNA sorting into cancer cell-derived extracellular vesicle subtypes. eLife.

[69] Wei H., Chen Q., Lin L., Sha C., Li T., Liu Y., Yin X., Xu Y., Chen L., Gao W. (2021). Regulation of exosome production and cargo sorting. Int. J. Biol. Sci..

[70] McKenzie A.J., Hoshino D., Hong N.H., Cha D.J., Franklin J.L., Coffey R.J., Patton J.G., Weaver A.M. (2016). KRAS-MEK signaling controls Ago2 sorting into exosomes. Cell Rep..

[71] Guduric-Fuchs J., O’Connor A., Camp B., O’Neill C.L., Medina R.J., Simpson D.A. (2012). Selective extracellular vesicle-mediated export of an overlapping set of microRNAs from multiple cell types. BMC Genom..

[72] Zhang H., Deng T., Liu R., Ning T., Yang H., Liu D., Zhang Q., Lin D., Ge S., Bai M. (2020). CAF secreted miR-522 suppresses ferroptosis and promotes acquired chemo-resistance in gastric cancer. Mol. Cancer.

[73] Gao T., Shu J., Cui J. (2018). A systematic approach to RNA-associated motif discovery. BMC Genom..

[74] Xu Y.-F., Hannafon B.N., Khatri U., Gin A., Ding W.-Q. (2019). The origin of exosomal miR-1246 in human cancer cells. RNA Biol..

[75] Villarroya-Beltri C., Gutiérrez-Vázquez C., Sánchez-Cabo F., Pérez-Hernández D., Vázquez J., Martin-Cofreces N., Martinez-Herrera D.J., Pascual-Montano A., Mittelbrunn M., Sánchez-Madrid F. (2013). Sumoylated hnRNPA2B1 controls the sorting of miRNAs into exosomes through binding to specific motifs. Nat. Commun..

[76] Tietje A., Maron K.N., Wei Y., Feliciano D.M. (2014). Cerebrospinal fluid extracellular vesicles undergo age dependent declines and contain known and novel non-coding RNAs. PLoS ONE.

[77] Santangelo L., Giurato G., Cicchini C., Montaldo C., Mancone C., Tarallo R., Battistelli C., Alonzi T., Weisz A., Tripodi M. (2016). The RNA-binding protein SYNCRIP is a component of the hepatocyte exosomal machinery controlling microRNA sorting. Cell Rep..

[78] Hobor F., Dallmann A., Ball N.J., Cicchini C., Battistelli C., Ogrodowicz R.W., Christodoulou E., Martin S.R., Castello A., Tripodi M. (2018). A cryptic RNA-binding domain mediates Syncrip recognition and exosomal partitioning of miRNA targets. Nat. Commun..

[79] Cooks T., Pateras I.S., Jenkins L.M., Patel K.M., Robles A.I., Morris J., Forshew T., Appella E., Gorgoulis V.G., Harris C.C. (2018). Mutant p53 cancers reprogram macrophages to tumor supporting macrophages via exosomal miR-1246. Nat. Commun..

[80] Mittelbrunn M., Gutiérrez-Vázquez C., Villarroya-Beltri C., González S., Sánchez-Cabo F., González M.Á., Bernad A., Sánchez-Madrid F. (2011). Unidirectional transfer of microRNA-loaded exosomes from T cells to antigen-presenting cells. Nat. Commun..

[81] Kosaka N., Iguchi H., Hagiwara K., Yoshioka Y., Takeshita F., Ochiya T. (2013). Neutral sphingomyelinase 2 (nSMase2)-dependent exosomal transfer of angiogenic microRNAs regulate cancer cell metastasis. J. Biol. Chem..

[82] Kosaka N., Iguchi H., Yoshioka Y., Takeshita F., Matsuki Y., Ochiya T. (2010). Secretory mechanisms and intercellular transfer of microRNAs in living cells. J. Biol. Chem..

[83] Wei J.x., Lv L.h., Wan Y.l., Cao Y., Li G.l., Lin H.m., Zhou R., Shang C.z., Cao J., He H. (2015). Vps4A functions as a tumor suppressor by regulating the secretion and uptake of exosomal microRNAs in human hepatoma cells. Hepatology.

[84] Lin F., Zeng Z., Song Y., Li L., Wu Z., Zhang X., Li Z., Ke X., Hu X. (2019). YBX-1 mediated sorting of miR-133 into hypoxia/reoxygenation-induced EPC-derived exosomes to increase fibroblast angiogenesis and MEndoT. Stem Cell Res. Ther..

[85] Shurtleff M.J., Temoche-Diaz M.M., Karfilis K.V., Ri S., Schekman R. (2016). Y-box protein 1 is required to sort microRNAs into exosomes in cells and in a cell-free reaction. eLife.

[86] Lu P., Li H., Li N., Singh R.N., Bishop C.E., Chen X., Lu B. (2017). MEX3C interacts with adaptor-related protein complex 2 and involves in miR-451a exosomal sorting. PLoS ONE.

[87] Dickman C.T., Lawson J., Jabalee J., MacLellan S.A., LePard N.E., Bennewith K.L., Garnis C. (2017). Selective extracellular vesicle exclusion of miR-142-3p by oral cancer cells promotes both internal and extracellular malignant phenotypes. Oncotarget.

[88] Teng Y., Ren Y., Hu X., Mu J., Samykutty A., Zhuang X., Deng Z., Kumar A., Zhang L., Merchant M.L. (2017). MVP-mediated exosomal sorting of miR-193a promotes colon cancer progression. Nat. Commun..

[89] Janas T., Janas M.M., Sapoń K., Janas T. (2015). Mechanisms of RNA loading into exosomes. FEBS Lett..

[90] Momose F., Seo N., Akahori Y., Sawada S.-I., Harada N., Ogura T., Akiyoshi K., Shiku H. (2016). Guanine-rich sequences are a dominant feature of exosomal microRNAs across the mammalian species and cell types. PLoS ONE.

[91] Bolukbasi M.F., Mizrak A., Ozdener G.B., Madlener S., Ströbel T., Erkan E.P., Fan J.-B., Breakefield X.O., Saydam O. (2012). miR-1289 and “Zipcode”-like sequence enrich mRNAs in microvesicles. Mol. Ther.-Nucleic Acids.

[92] Squadrito M.L., Baer C., Burdet F., Maderna C., Gilfillan G.D., Lyle R., Ibberson M., De Palma M. (2014). Endogenous RNAs modulate microRNA sorting to exosomes and transfer to acceptor cells. Cell Rep..

[93] Cha D.J., Franklin J.L., Dou Y., Liu Q., Higginbotham J.N., Beckler M.D., Weaver A.M., Vickers K., Prasad N., Levy S. (2015). KRAS-dependent sorting of miRNA to exosomes. eLife.

[94] Jin W., Shi J., Liu M. (2019). Overexpression of miR-671-5p indicates a poor prognosis in colon cancer and accelerates proliferation, migration, and invasion of colon cancer cells. Onco Targets.

[95] Xu J., Zhao J., Zhang R. (2017). Prognostic significance of serum miR-193b in colorectal cancer. Int. J. Clin. Exp. Pathol..

[96] Li J., Peng W., Yang P., Chen R., Gu Q., Qian W., Ji D., Wang Q., Zhang Z., Tang J. (2020). MicroRNA-1224-5p Inhibits Metastasis and Epithelial-Mesenchymal Transition in Colorectal Cancer by Targeting SP1-Mediated NF-κB Signaling Pathways. Front. Oncol..

[97] Fujino Y., Takeishi S., Nishida K., Okamoto K., Muguruma N., Kimura T., Kitamura S., Miyamoto H., Fujimoto A., Higashijima J. (2017). Downregulation of microRNA-100/microRNA-125b is associated with lymph node metastasis in early colorectal cancer with submucosal invasion. Cancer Sci..

[98] Danac J.M.C., Uy A.G.G., Garcia R.L. (2021). Exosomal microRNAs in colorectal cancer: Overcoming barriers of the metastatic cascade (Review). Int. J. Mol. Med..

[99] Niu Z.-Y., Li W.-L., Jiang D.-L., Li Y.-S., Xie X.-J. (2018). Mir-483 inhibits colon cancer cell proliferation and migration by targeting TRAF1. Kaohsiung J. Med. Sci..

[100] Zhu X., Luo X., Song Z., Jiang S., Long X., Gao X., Xie X., Zheng L., Wang H. (2021). miR-188-5p promotes oxaliplatin resistance by targeting RASA1 in colon cancer cells. Oncol. Lett..

[101] Yu Y., Lu X., Yang C., Yin F. (2020). Long Noncoding RNA LINC00173 Contributes to the Growth, Invasiveness and Chemo-Resistance of Colorectal Cancer Through Regulating miR-765/PLP2 Axis. Cancer Manag. Res..

[102] Zhang J., Fei B., Wang Q., Song M., Yin Y., Zhang B., Ni S., Guo W., Bian Z., Quan C. (2014). MicroRNA-638 inhibits cell proliferation, invasion and regulates cell cycle by targeting tetraspanin 1 in human colorectal carcinoma. Oncotarget.

[103] Li P., Cai J.-X., Han F., Wang J., Zhou J.-J., Shen K.-W., Wang L.-H. (2020). Expression and significance of miR-654-5p and miR-376b-3p in patients with colon cancer. World J. Gastrointest. Oncol..

[104] Lai H., Zhang J., Zuo H., Liu H., Xu J., Feng Y., Lin Y., Mo X. (2020). Overexpression of miR-17 is correlated with liver metastasis in colorectal cancer. Medicine.

[105] Yang J., Zhao H., Xin Y., Fan L. (2014). MicroRNA-198 Inhibits Proliferation and Induces Apoptosis of Lung Cancer Cells Via Targeting FGFR1. J. Cell. Biochem..

[106] Cui F.C., Chen Y., Wu X.Y., Hu M., Qin W.S. (2020). MicroRNA-493-5p suppresses colorectal cancer progression via the PI3K-Akt-FoxO3a signaling pathway. Eur. Rev. Med. Pharmacol. Sci..

[107] Chai B., Guo Y., Cui X., Liu J., Suo Y., Dou Z., Li N. (2019). MiR-223-3p promotes the proliferation, invasion and migration of colon cancer cells by negative regulating PRDM1. Am. J. Transl. Res..

[108] Vishnubalaji R., Hamam R., Yue S., Al-Obeed O., Kassem M., Liu F.-F., Aldahmash A., Alajez N.M. (2016). MicroRNA-320 suppresses colorectal cancer by targeting SOX4, FOXM1, and FOXQ1. Oncotarget.

[109] Liu X., Chen X., Zeng K., Xu M., He B., Pan Y., Sun H., Pan B., Xu X., Xu T. (2018). DNA-methylation-mediated silencing of miR-486-5p promotes colorectal cancer proliferation and migration through activation of PLAGL2/IGF2/β-catenin signal pathways. Cell Death Dis..

[110] Baassiri A., Nassar F., Mukherji D., Shamseddine A., Nasr R., Temraz S. (2020). Exosomal Non Coding RNA in LIQUID Biopsies as a Promising Biomarker for Colorectal Cancer. Int. J. Mol. Sci..

[111] Bach D.H., Hong J.Y., Park H.J., Lee S.K. (2017). The role of exosomes and miRNAs in drug-resistance of cancer cells. Int. J. Cancer.

[112] Guo Q.-R., Wang H., Yan Y.-D., Liu Y., Su C.-Y., Chen H.-B., Yan Y.-Y., Adhikari R., Wu Q., Zhang J.-Y. (2020). The role of exosomal microRNA in cancer drug resistance. Front. Oncol..

[113] Lopes-Rodrigues V., Seca H., Sousa D., Sousa E., Lima R.T., Vasconcelos M.H. (2014). The network of P-glycoprotein and microRNAs interactions. Int. J. Cancer.

[114] Liu T., Zhang X., Du L., Wang Y., Liu X., Tian H., Wang L., Li P., Zhao Y., Duan W. (2019). Exosome-transmitted miR-128-3p increase chemosensitivity of oxaliplatin-resistant colorectal cancer. Mol. Cancer.

[115] Holohan C., Van Schaeybroeck S., Longley D.B., Johnston P.G. (2013). Cancer drug resistance: An evolving paradigm. Nat. Rev. Cancer.

[116] Goldstein M., Kastan M.B. (2015). The DNA damage response: Implications for tumor responses to radiation and chemotherapy. Annu. Rev. Med..

[117] Jackson S.P., Bartek J. (2009). The DNA-damage response in human biology and disease. Nature.

[118] Nakajima G., Hayashi K., Xi Y., Kudo K., Uchida K., Takasaki K., Yamamoto M., Ju J. (2006). Non-coding microRNAs hsa-let-7g and hsa-miR-181b are associated with chemoresponse to S-1 in colon cancer. Cancer Genom.-Proteom..

[119] Wang S., Zeng Y., Zhou J.M., Nie S.L., Peng Q., Gong J., Huo J.R. (2016). MicroRNA-1246 promotes growth and metastasis of colorectal cancer cells involving CCNG2 reduction. Mol. Med. Rep..

[120] Hasegawa S., Eguchi H., Nagano H., Konno M., Tomimaru Y., Wada H., Hama N., Kawamoto K., Kobayashi S., Nishida N. (2014). MicroRNA-1246 expression associated with CCNG2-mediated chemoresistance and stemness in pancreatic cancer. Br. J. Cancer.

[121] Li X.J., Ren Z.J., Tang J.H., Yu Q. (2017). Exosomal MicroRNA MiR-1246 promotes cell proliferation, invasion and drug resistance by targeting CCNG2 in breast cancer. Cell. Physiol. Biochem..

[122] Hanahan D., Weinberg R.A. (2011). Hallmarks of cancer: The next generation. Cell.

[123] Yoshida G.J. (2015). Metabolic reprogramming: The emerging concept and associated therapeutic strategies. J. Exp. Clin. Cancer Res..

[124] Gatenby R.A., Gillies R.J. (2004). Why do cancers have high aerobic glycolysis?. Nat. Rev. Cancer.

[125] Hirschhaeuser F., Sattler U.G., Mueller-Klieser W. (2011). Lactate: A metabolic key player in cancer. Cancer Res..

[126] Song B., Wang Y., Xi Y., Kudo K., Bruheim S., Botchkina G.I., Gavin E., Wan Y., Formentini A., Kornmann M. (2009). Mechanism of chemoresistance mediated by miR-140 in human osteosarcoma and colon cancer cells. Oncogene.

